# Supplementary material for: Collaborative scheduling of dual-trolley quay cranes and AGVs via speed-control strategy
Source: PLoS One. 2025 Dec 29;20(12):e0339585. doi: 10.1371/journal.pone.0339585 (PMC12747412; doi:10.1371/journal.pone.0339585)
Supplement: S1 File — (DOCX) [file pone.0339585.s001.docx]

Collaborative Scheduling of Dual-trolley Quay Cranes and AGVs via Speed-Control Strategy

**S1-Models**

1、Layout of dual-trolley QC automated terminal [1].


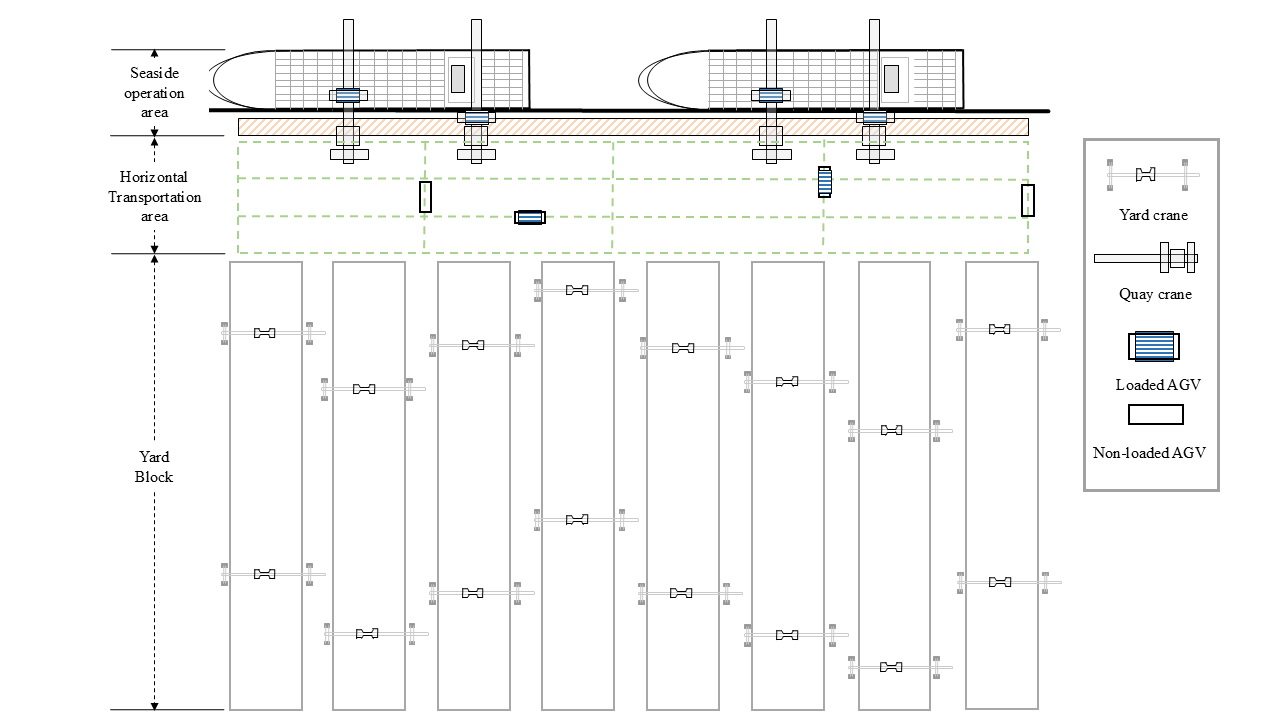


**Fig. S1.** Layout of dual-trolley QC automated terminal.

2、Network Topology

| **Node** | 1 | 2 | 3 | 4 | 5 | 6 | 7 | 8 | 9 |
| --- | --- | --- | --- | --- | --- | --- | --- | --- | --- |
| **Coordinate** | (0,0) | (20,0) | (40,0) | (60,0) | (80,0) | (100,0) | (120,0) | (140,0) | (160,0) |
| **Node** | 10 | 11 | 12 | 13 | 14 | 15 | 16 | 17 | 18 |
| **Coordinate** | (0,20) | (20,20) | (40, 20) | (60, 20) | (80,20) | (100,20) | (120,20) | (140,20) | (160,20) |
| **Node** | 19 | 20 | 21 | 22 | 23 | 24 | 25 | 26 | 27 |
| **Coordinate** | (0,40) | (20,40) | (40,40) | (60,40) | (80,40) | (100,40) | (120,40) | (140,40) | (160,40) |
| **Node** | 28 | 29 | 30 | 31 | 32 | 33 | 34 | 35 | 36 |
| **Coordinate** | (0,60) | (20,60) | (40,60) | (60,60) | (80,60) | (100,60) | (120,60) | (140,60) | (160,60) |

**Table. S1.** Network Topology

3、Task Assignment and Routes

| **AGV Index** | **Task Route** | **Travel Route** |
| --- | --- | --- |
| **1** | 2-35 | 2-3-4-5-6-7-8-9-18-27-36-35 |
|  | 35-8 | 35-34-25-16-7-8 |
|  | 8-29 | 8-9-18-17-16-15-14-13-12-21-30-29 |
|  | 29-2 | 29-28-19-10-1-2 |
| **2** | 8-29 | 8-9-18-27-36-35-34-33-32-31-30-29 |
|  | 29-2 | 29-28-19-10-1-2 |
|  | 2-35 | 2-3-4-5-6-7-8-9-18-27-36-35 |
|  | 35-8 | 35-34-25-16-7-8 |
| **3** | 29-2 | 29-28-19-10-1-2 |
|  | 2-35 | 2-3-12-21-22-23-24-25-26-27-36-35 |
|  | 35-8 | 35-34-25-16-7-8 |
|  | 8-35 | 8-9-18-27-36-35 |
| **4** | 35-8 | 35-34-25-16-7-8 |
|  | 8-29 | 8-9-18-27-36-35-34-33-32-31-30-29 |
|  | 29-2 | 29-28-19-10-1-2 |
|  | 2-29 | 2-3-12-21-30-29 |

4、Parameters, descriptions, and values in the case study

| **Parameter** | **Description** | **Value [1]** |
| --- | --- | --- |
| **L** | Length of an AGV. | 6m |
| **l** | Length of each grid path segment. | 20m |
| $\boldsymbol{V}_{\mathbf{0}}$ | Constant speed during normal AGV operation. | 4m/s |
| $\boldsymbol{V}_{\mathbf{2}}$ | Maximum safety-compliant driving speed. | 5m/s |
| $\boldsymbol{L}_{\boldsymbol{s}}$ | Minimum safety distance between AGVs. | 4m |
| **a** | The AGV acceleration. | 1m/s^2^ |
| $\boldsymbol{v}_{\boldsymbol{t}}$ | The main trolley's operational speed. | 80s/move |
| $\boldsymbol{v}_{\boldsymbol{g}}$ | ​The gantry trolley’s operational speed. | 60s/move |
| **c** | Maximum containers stored of the transfer platform. | 2 |
| $\boldsymbol{t}_{\boldsymbol{YC}}$ | The YC operation time. | 10s |

**Table. S1.** Parameters, descriptions, and values in the case study.

| AGV1 | | | | | | | | | | |
| --- | --- | --- | --- | --- | --- | --- | --- | --- | --- | --- |
| Time(s) | 0 | 4 | 22.23 | 23.6 | 27.4 | 28.77 | 61.77 | 65.77 | 75.77 | 79.77 |
| Velocity(m/s) | 0 | 4 | 4 | 2.63 | 2.63 | 4 | 4 | 0 | 0 | 4 |
| Time(s) | 105.77 | 109.77 | 169.77 | 173.77 | 229.77 | 233.77 | 243.77 | 247.77 | 273.77 | 277.77 |
| Velocity(m/s) | 4 | 0 | 0 | 4 | 4 | 0 | 0 | 4 | 4 | 0 |

| AGV2 | | | | | | | | | | |
| --- | --- | --- | --- | --- | --- | --- | --- | --- | --- | --- |
| Time(s) | 0 | 4 | 60 | 64 | 74 | 78 | 104 | 108 | 168 | 172 |
| Velocity(m/s) | 0 | 4 | 4 | 0 | 0 | 4 | 4 | 0 | 0 | 4 |
| Time(s) | 190.86 | 192.09 | 195.07 | 196.3 | 229.3 | 233.3 | 243.3 | 247.3 | 273.3 | 277.3 |
| Velocity(m/s) | 4 | 2.77 | 2.77 | 4 | 4 | 0 | 0 | 4 | 4 | 0 |

| AGV3 | | | | | | | | | | | | |
| --- | --- | --- | --- | --- | --- | --- | --- | --- | --- | --- | --- | --- |
| Time(s) | 0 | 4 | 30 | 34 | 94 | 98 | 154 | 158 | 168 | 172 | 188.88 | 189.88 |
| Velocity(m/s) | 0 | 4 | 4 | 0 | 0 | 4 | 4 | 0 | 0 | 4 | 4 | 5 |
| Time(s) | 190.86 | 192.09 | 195.07 | 196.3 | 229.3 | 233.3 | 243.3 | 247.3 | 273.3 | 277.3 |  |  |
| Velocity(m/s) | 4 | 2.77 | 2.77 | 4 | 4 | 0 | 0 | 4 | 4 | 0 |  |  |

| AGV4 | | | | | | | | | | |
| --- | --- | --- | --- | --- | --- | --- | --- | --- | --- | --- |
| Time(s) | 0 | 4 | 25.88 | 26.88 | 27.78 | 28.78 | 29.53 | 33.53 | 93.53 | 97.53 |
| Velocity(m/s) | 0 | 4 | 4 | 5 | 5 | 4 | 4 | 0 | 0 | 4 |
| Time(s) | 157.53 | 167.53 | 171.53 | 197.53 | 201.53 | 261.53 | 265.53 | 291.53 | 295.53 | 157.53 |
| Velocity(m/s) | 0 | 0 | 4 | 4 | 0 | 0 | 4 | 4 | 0 | 0 |

Speed control strategy occupancy diagram

| Spatial conflict  point | Status and duration(s) | | | | | | | | | | | |
| --- | --- | --- | --- | --- | --- | --- | --- | --- | --- | --- | --- | --- |
| 7 | Unoccupied | Occupied  by AGV4 | Unoccupied | Occupied  by AGV1 | Unoccupied | Occupied  by AGV1 | Unoccupied | Occupied  by AGV3 | Unoccupied | Occupied  by AGV2 | Unoccupied | Occupied  by AGV2 |
|  | 26.875 | 1.2 | 0.695 | 1.5 | 72.5 | 1.5 | 90.13 | 1.2 | 0.7 | 1.5 | 72.5 | 1.5 |
| 12 | Unoccupied | Occupied  by AGV3 | Unoccupied | Occupied  by AGV1 | Unoccupied | Occupied  by AGV4 |  |  |  |  |  |  |
|  | 106 | 1.5 | 104.27 | 1.5 | 60.255 | 1.5 |  |  |  |  |  |  |
| 16 | Unoccupied | Occupied  by AGV4 | Unoccupied | Occupied  by AGV1 | Unoccupied | Occupied  by AGV3 | Unoccupied | Occupied  by AGV1 | Unoccupied | Occupied  by AGV2 |  |  |
|  | 22 | 1.5 | 74.27 | 1.5 | 90.605 | 1.2 | 0.695 | 1.5 | 72.03 | 1.5 |  |  |
| 25 | Unoccupied | Occupied  by AGV4 | Unoccupied | Occupied  by AGV1 | Unoccupied | Occupied  by AGV3 | Unoccupied | Occupied  by AGV3 | Unoccupied | Occupied  by AGV2 |  |  |
|  | 12 | 1.5 | 74.27 | 1.5 | 46.73 | 1.5 | 42.5 | 1.5 | 73.8 | 1.5 |  |  |
| 27 | Unoccupied | Occupied  by AGV2 | Unoccupied | Occupied  by AGV1 | Unoccupied | Occupied  by AGV4 | Unoccupied | Occupied  by AGV3 | Unoccupied | Occupied  by AGV2 | Unoccupied | Occupied  by AGV3 |
|  | 22 | 1.5 | 30.27 | 1.5 | 60.255 | 1.5 | 28.975 | 1.5 | 73.8 | 1.5 | 60.25 | 1.5 |
| 30 | Unoccupied | Occupied  by AGV2 | Unoccupied | Occupied  by AGV4 | Unoccupied | Occupied  by AGV1 | Unoccupied | Occupied  by AGV4 |  |  |  |  |
|  | 57 | 1.5 | 92.025 | 1.5 | 74.745 | 1.5 | 60.255 | 1.5 |  |  |  |  |
| 2 | Unoccupied | Occupied  by AGV3 | Unoccupied | Occupied  by AGV2 | Unoccupied | Occupied  by AGV4 | Unoccupied | Occupied  by AGV1 |  |  |  |  |
|  | 34 | 60 | 14 | 60 | 33.525 | 60 | 16.245 | 30 |  |  |  |  |
| 8 | Unoccupied | Occupied  by AGV4 | Unoccupied | Occupied  by AGV1 | Unoccupied | Occupied  by AGV3 | Unoccupied | Occupied  by AGV2 |  |  |  |  |
|  | 33.525 | 60 | 16.245 | 60 | 31.28 | 60 | 16.25 | 30 |  |  |  |  |
| 29 | Unoccupied | Occupied  by AGV2 | Unoccupied | Occupied  by AGV4 | Unoccupied | Occupied  by AGV1 | Unoccupied | Occupied  by AGV4 |  |  |  |  |
|  | 64 | 10 | 83.525 | 10 | 66.245 | 10 | 51.755 | 10 |  |  |  |  |
| 35 | Unoccupied | Occupied  by AGV1 | Unoccupied | Occupied  by AGV3 | Unoccupied | Occupied  by AGV2 | Unoccupied | Occupied  by AGV3 |  |  |  |  |
|  | 65.77 | 10 | 82.23 | 10 | 65.3 | 10 | 51.75 | 10 |  |  |  |  |

**References**

1. Yue, L.J. Research on Configuration and Scheduling of Double-trolley Quay Crane and AGV in Automated Container Terminal. Dalian Maritime University, 2020. DOI:10.26989/d.cnki.gdlhu.2020.001070.
