# Supplementary material for: Collaborative scheduling of dual-trolley quay cranes and AGVs via speed-control strategy
Source: PLoS One. 2025 Dec 29;20(12):e0339585. doi: 10.1371/journal.pone.0339585 (PMC12747412; doi:10.1371/journal.pone.0339585)
Supplement: Attachment — Submitted filename: Response to Reviewers.docx [file pone.0339585.s004.docx]

**Response to Reviewers**

**Manuscript Number:** PONE-D-25-20515

**Title:** Collaborative Scheduling of Dual-trolley Quay Cranes and AGVs via Speed-Control Strategy

Dear Editors and Reviewers,

We appreciate the opportunity to revise our manuscript titled " Collaborative Scheduling of Dual-trolley Quay Cranes and AGVs via Speed-Control Strategy" and are grateful for the insightful comments provided by the reviewers. Those comments are all valuable and very helpful for revising and improving our paper, as well as the important guiding significance to our researches. In the following, we have provided detailed responses to each of the reviewers' comments. Revised portion are marked in red in the paper. Additionally, we have conducted a comprehensive revision of the entire manuscript. In this response letter, the reviewers' comments are presented in italics, and our corresponding changes and additions to the manuscript are highlighted in highlight text. We have tried our best to make all the revisions clear, and we hope that the revised manuscript meets the requirements for publication.

**Reviewer #1:**

**Comments 1:** *The reviewer endorses thr publication of the present manuscript because the athour/s explores in a new way an old topic: conflits that can occur when an automatic guided vehicles reach the dual-trolley quay cranes (DQCs) promptly during automated terminal operations. This could lead to congestion on the transfer platform and diminish the operational efficiency of unmanned terminals. So, the author/s have proposed a cooperative scheduling approach integrating a speed control strategy for DQCs and AGVs demostrating that the proposed speed-control strategy effectively resolves conflicts while consuming less energy than traditional stopand-wait methods.*

**Response 1:** We sincerely appreciate your endorsement of our manuscript's value and your insightful review. Your keen understanding of our work is most encouraging. You have precisely captured the core of our research: the conflicts arising at the interaction between Automated Guided Vehicles (AGVs) and Dual-trolley Quay Cranes (DQCs) in automated terminal operations, and the consequent platform congestion and efficiency degradation. We fully concur with your summary. Indeed, as you pointed out, the integrated cooperative scheduling approach with a DQC speed control strategy was proposed to address this critical issue, aiming to resolve conflicts while enhancing overall system efficiency and reducing energy consumption. We are pleased that you recognized the novelty of our research and the effectiveness of our proposed strategy in conflict resolution and energy conservation.

**Reviewer #2:**

**Comments 1:***(Introduction) Introduction contains only one reference. Further explanation is necessary regarding problem description and research gap, along with an overview of industry trends and environment, and sufficient references. Ultimately, the research question and research objective are not logically derived and are not clearly articulated.*

**Response 1:** We would like to express our sincere gratitude to the reviewer for your insightful comments and valuable feedback. In the revised Introduction, two pertinent and high-quality references [2,3] have been appended to refine the literature review. Their substantive contributions are respectively discussed in lines 4–5 of the first paragraph and lines 3–4 of the third paragraph. These insertions consolidate the logical transition from industrial context to identified research gaps and, accordingly, enable the precise formulation of the research questions and objectives addressed herein.

**Comments 2:** *(Literature)*

*Reference [7] seems to be analogous to this study. In what way does it differ from [7]?*

*“Although the aforementioned static …… and other factors.” The limitations of existing studies are expressed too abstractly. It is necessary to list specific limitations. Also, why were the limitations not summarized in this way in other subsections?*

*“This study considers the transfer platform …… due to conflicts.” This paragraph should be placed in Introduction to emphasize how it differs from existing research.*

**Response 2:** We sincerely appreciate the valuable comment.

(1) This study addresses the collaborative scheduling of double-trolley quay cranes and AGVs. By introducing, for the first time, a speed-control mechanism that eliminates stopping while guaranteeing precise alignment with crane time windows, it constitutes a decisive advance in integrated scheduling and conflict-free operation. The present study differs substantially from Reference [7] in problem domain, core constraints, and optimization objectives; a detailed comparison is provided in Table below.

|  | Reference [7]  Quay crane scheduling with time windows... | Our paper  Collaborative Scheduling of Dual-trolley Quay Cranes and AGVs... |
| --- | --- | --- |
| Problem domain | Operation scheduling for a single automated quay crane (AQC) | Collaborative Scheduling of Dual-trolley Quay Cranes (DQC) and AGV |
| Core constraints | Container handling by quay cranes is subject to time window constraints. | The capacity limitation of the transfer platform is translated into time window constraints for the AGVs. |
| Optimization objectives | Minimizing makespan + energy consumption | Minimizing AGV energy consumption + maximizing time window satisfaction rate |
| Conflict taxonomy | None (single equipment, spatial conflicts not considered) | Multi-AGV spatial conflicts: node conflicts & catching-up conflicts |
| Conflict-resolution mechanism | None | Speed control strategy: high-priority AGVs accelerate, low-priority decelerate (non-stop) |

(2) Thank you for your valuable comment. We have incorporated a specific description of the limitations of static path planning research in the last paragraph of Section 3 of this chapter. The reason for not summarizing limitations in the same manner across other sections is that this paragraph focuses on providing a comprehensive assessment of the current research landscape, aiming to highlight common bottlenecks, whereas other sections are primarily dedicated to specific models or algorithms, with their respective limitations addressed separately within their experimental analyses and discussions.

（3）The paragraph has been moved to the end of the introduction as a lead-in to the following sections.

**Comments 3:** *(Model)Is the application of the Dijkstra algorithm to the new scheduling model a distinguishing feature of this study? This is not clearly expressed. Has this algorithm not been used before? Is there any reason to use this algorithm?*

**Response 3:** Thank you for your thoughtful comments. We have added a description in lines 2–4 of the final paragraph of Section 3.1, Chapter 3, highlighting the critical role and inherent advantages of the Dijkstra's algorithm in AGV path planning, thereby justifying its selection.

**Comments 4:***(Case study)This section has the most significant issues with this study. What are the references for the parameter values in Table 2? Scheduling was performed using values in Table 3; however, since this is a static approach, the model is not validated. Appropriate distributions should be assumed for each variable, and a dynamic approach should be repeated several hundreds or thousands of times to validate the model. From the content in Case Study Section, it is not possible to conclude based on Table 5.*

*Parking strategy is not mentioned in the former sections; however, it is significantly addressed following Fig. 8.*

**Response 4:** (1) Thank you for your valuable suggestions. We have revised the manuscript accordingly. The terminal layout in this study and the parameter values listed in Table 2 are based on the corresponding data of Shanghai Yangshan Port , according to the additional reference [37]. This matter has also been addressed in lines 1–2 of the third paragraph of Chapter 4.

(2) In response to the reviewer's suggestions, we have added a new subsection at the end of Chapter 4 to validate the proposed model. Considering the uncertainties in terminal operations, we designed appropriate probability distributions for key variables and conducted 1,000 Monte Carlo simulation runs. A comparative analysis between the proposed speed control strategy and the conventional stop-and-wait strategy was performed, demonstrating the superiority and robustness of our model.

(3) In the second paragraph at the beginning of Chapter 4, we have added an explanation for the rationale behind comparing speed control strategies, thereby introducing the subsequent case study's comparison with stop-and-wait strategies and enhancing the logical coherence of the paper.

**Comments 5:** *(Conclusion)What is the practical implication of the model?*

*Are there any limitations to this study?*

**Response 5:** In the second paragraph of Chapter 5, we have added an elaboration on the practical implications of this research, highlighting its significance in resolving AGV path conflicts and enhancing collaborative scheduling efficiency in automated terminals. Furthermore, in paragraphs 3-6 of the same chapter, the limitations of the study are discussed, primarily focusing on the insufficient consideration of the impact of payload variations on AGV energy consumption and AGV charging requirements.

**Comments 6:** *Minor issues*

*Abbreviations should be defined with their full names at the first time.*

*English editing service is necessary.*

*What is the difference between model assumptions 3 and 6?*

*The explanations of notations l1 and l2 are not intuitive.*

**Response 5:** (1) We sincerely thank the reviewer for this important reminder. We have carefully reviewed the entire manuscript and ensured that all abbreviations are defined with their full names upon first occurrence.

(2) Thank you for pointing out the potential overlap in these assumptions. We clarify the distinction as follows:

Assumption 3 states that “AGVs travel along unidirectional single-lane paths.” This refers to the physical layout of the road network, meaning that each lane is designed for one-way traffic, which helps reduce head-on conflicts.

Assumption 6 states that “All AGVs are restricted to unidirectional movement without reverse motion.” This refers to the operational rule for AGV behavior, meaning that AGVs are not allowed to reverse during travel, which simplifies motion control and conflict resolution.

(3) To improve clarity, we have revised the explanations of $l_{1}$ and $l_{2}$ in the **Notations and Explanations** section (Table 1) as follows:

- $l_{1}$: **Deceleration distance** — the distance an AGV travels while decelerating from $V_{0}$ to $V_{1}$.
- $l_{2}$: **Acceleration distance** — the distance an AGV travels while accelerating from $V_{0}$ to $V_{2}$.

**Reviewer #3:**

**Comments 1:**

*Title and Abstract*

*The title is appropriate and indicates the main message of the paper No changes necessary. The abstract is understandable by a non-specialist reader No changes necessary*

*Introduction：Does the introduction provide sufficient background information for readers not in the immediate field to understand the problem/hypotheses?*

*Yes*

*Are the reasons for performing the study clearly defined?*

*yes*

*Are the study objectives clearly defined?*

*Yes*

*Material and methods and result*

*Are the methods used appropriate to the aims of the study?*

*• yes*

*Results/Statistics*

*The results are clearly explained and presented in an appropriate*

*Discussion*

*The findings of the study are properly described in the context of the published literature.*

*The conclusions address the aim of the study No changes necessary*

*Literature Cited*

*The literature cited is appropriate, recent and balanced*

*Significance and Novelty*

*The claims are sufficiently novel to warrant publication No changes necessary*

*Thanks for such work,*

**Response 1:** We sincerely appreciate your positive evaluation and comprehensive positive feedback on our manuscript titled "Collaborative Scheduling of Dual-trolley Quay Cranes and AGVs via Speed-Control Strategy". We are delighted that you have endorsed all aspects of our work—including the title, abstract, introduction, methodology, results, discussion, and literature review—without suggesting any revisions. Your overall approval of the paper's structure, logical flow, and scholarly rigor is a tremendous encouragement to our research team. We are particularly honored by your specific acknowledgment that the claims of our study are "sufficiently novel to warrant publication."
