# Supplementary material for: Collaborative scheduling of dual-trolley quay cranes and AGVs via speed-control strategy
Source: PLoS One. 2025 Dec 29;20(12):e0339585. doi: 10.1371/journal.pone.0339585 (PMC12747412; doi:10.1371/journal.pone.0339585)
Supplement: Attachment — Submitted filename: Response_to_Reviewers_auresp_2.docx [file pone.0339585.s005.docx]

**Response to Reviewers**

**Manuscript Number:** PONE-D-25-20515

**Title:** Collaborative Scheduling of Dual-trolley Quay Cranes and AGVs via Speed-Control Strategy

Dear Editors and Reviewers,

We would like to once again appreciate your time and consideration of our manuscript, as well as for the additional thoughtful and constructive comments you have provided. Following your valuable suggestions, we have carefully revised our manuscript. All modifications made in the text have been highlighted and are pointed out in the response below. We hope that these further revisions will improve the quality of our manuscript to make it sufficient for further consideration, and we are deeply grateful for your guidance and patience throughout this process.

Thank you once again for your time and expertise, and we are looking forward to hearing from you soon.

Best wishes,

Yours sincerely,

Fang Yan

**Reviewer #2:**

**Comment 1:** *AGV and DQC should be defined in the manuscript first, not in the Abstract.*

**Response 1:** Thank you for raising this important point regarding the definition of technical abbreviations. In response to your comment, we have revised the manuscript to ensure that all abbreviations are properly defined upon their first occurrence in the main text. Specifically, the abbreviation "AGV" is now defined when it first appears in the second-to-last line of the first paragraph in Chapter 1. Similarly, the abbreviation "DQC" is defined at its first occurrence in the first line of the third paragraph of Chapter 1. We believe these revisions improve the clarity and academic rigor of the manuscript. We believe these modifications will enhance the overall quality and standardization of the manuscript, ensuring it conforms to academic standards and meets the journal's expectations

**Comment 2:***Newly added sentence "AGVs significant types of equipment in an automated terminals, which can walk[27]along a pre-set guidance path to complete a series of horizontal transport operations [2]." is not complete I think. Did you get the language editing service? You should attach the certificate of English editing.*

**Response 2:**We sincerely thank the reviewer for the careful reading and valuable comments. We deeply apologize for the incomplete and awkward sentence and this sentence has now been revised for clarity and grammatical correctness. The revised version appears in Section 1, Paragraph 1 (now at the end of that paragraph). In addition, we have carefully proofread the entire manuscript to eliminate similar issues and improve overall language quality.

Regarding language editing, we did not use a professional language editing service. However, to ensure the linguistic accuracy and fluency of this paper, we invited a professor from the UK who specializes in this field to review and polish the language throughout the manuscript. Although we cannot provide a formal certificate from a commercial editing service, we believe the professor's academic background and native English proficiency guarantee the quality of language in this submission. If further proof is needed, we would be happy to provide relevant supporting information or a brief statement from the professor upon request.

Thank you again for your helpful suggestion.

**Comment 3:** *Figure 10 is not relevantly described. You may find errors.*

**Response 3:**Thank you for pointing out the insufficiency in the description of Figure 10. In response to your comment, we have provided a detailed explanation of Figure 10 in Section 4.4, lines 1–2, which clearly demonstrates that the speed control strategy proposed in this paper outperforms the conventional stop-and-wait strategy in terms of the maximum completion time. This comparison is intended to highlight the advantages of the proposed method.
